# Genetic ancestry is related to potential sources of breast cancer health disparities among Colombian women

**DOI:** 10.1371/journal.pone.0306037

**Published:** 2024-06-27

**Authors:** Laura Rey-Vargas, Lina María Bejarano-Rivera, Silvia J. Serrano-Gómez

**Affiliations:** 1 National Cancer Institute, Cancer Biology Research Group, Bogotá, D.C, Colombia; 2 Doctoral Program in Biological Sciences, Pontificia Universidad Javeriana, Bogotá, D.C, Colombia; 3 National Cancer Institute, Research Support and Follow-Up Group, Bogotá, D.C, Colombia; Universidade Federal de Minas Gerais, BRAZIL

## Abstract

Breast cancer health disparities are linked to clinical-pathological determinants, socioeconomic inequities, and biological factors such as genetic ancestry. These factors collectively interact in complex ways, influencing disease behavior, especially among highly admixed populations like Colombians. In this study, we assessed contributing factors to breast cancer health disparities according to genetic ancestry in Colombian patients from a national cancer reference center. We collected non-tumoral paraffin embedded (FFPE) blocks from 361 women diagnosed with breast cancer at the National Cancer Institute (NCI) to estimate genetic ancestry using a 106-ancestry informative marker (AIM) panel. Differences in European, Indigenous American (IA) and African ancestry fractions were analyzed according to potential sources of breast cancer health disparities, like etiology, tumor-biology, treatment administration, and socioeconomic-related factors using a Kruskal–Wallis test. Our analysis revealed a significantly higher IA ancestry among overweight patients with larger tumors and those covered by a subsidized health insurance. Conversely, we found a significantly higher European ancestry among patients with smaller tumors, residing in middle-income households, and affiliated to the contributory health regime, whereas a higher median of African ancestry was observed among patients with either a clinical, pathological, or stable response to neoadjuvant treatment. Altogether, our results suggest that the genetic legacy among Colombian patients, measured as genetic ancestry fractions, may be reflected in many of the clinical-pathological variables and socioeconomic factors that end up contributing to health disparities for this disease.

## Introduction

Health disparities in breast cancer have been defined in multiple ways, englobing aspects related to socioeconomic inequities, lack of access to screening protocols, poor quality of cancer care, along with differences in the presentation of several risk factors among populations, ultimately leading to differences in breast cancer incidence and mortality rates, as well as in the disease outcome among women with different race/ethnicities [1–3]. The latest 2022 American Cancer Society report revealed the highest breast cancer incidence in non-Hispanic White women (132.5 per 100.000), followed by non-Hispanic Blacks (127.1 per 100.000), American Indian/Alaska Natives (110.5 per 100.000), Asian/Pacific Islanders (98.8 per 100.000), and in the last place Hispanic/Latina women (96.3 per 100.000) [4]. In terms of mortality, non-Hispanic Black women presented the highest mortality rates among all population groups (28.0 per 100.000), followed by non-Hispanic Whites (19.9 per 100.000), American Indian/Alaska Natives (17.8 per 100.000), Hispanic/Latinas (13.7 per 100.000), and lastly Asian/Pacific Islanders women (11.7 per 100.000).

Recent studies have explored genetic ancestry as a biological factor that has been associated with differences in breast cancer risk and its phenotype, contributing this way to health disparities [5,6]. For instance, it has been reported that women with higher European component present higher odds for the disease [7,8], compared to women with higher Indigenous American (IA) ancestry [9], whereas women with greater African ancestry have higher odds for breast cancer-specific mortality [10,11]. On the other hand, differences in the prevalence of intrinsic subtypes between population groups have also been reported [12,13]. For example, luminal A breast tumors are more prevalent in women with higher European ancestry, whereas the triple-negative (TN) subtype is more frequently observed in women with a higher African component, and greater fractions of IA ancestry have been associated with higher odds for HER2-positive tumors [6,10,14]. These differences have also been associated to the differences in the disease outcome between women with different genetic ancestry fractions [15].

Genetic ancestry, as a variable that is objectively measured by assessing the differences in genome-allele frequencies of multiple polymorphisms among ancestral populations [15,16], might be associated to breast cancer health disparities. However, genetic ancestry can also be understood as a reflection of the human migration process and the Americas’ colonization history, and therefore, it can mirror the degree of poverty, racial/ethnic discrimination, and other socioeconomic determinants that also contribute to breast cancer health disparities among populations [17,18]. Other factors related to breast cancer health disparities include etiology, tumor biology, and treatment-related clinical-pathological variables. The correlation between socioeconomic and clinical-pathological determinants entails complex interactions, and adding genetic ancestry as a biological contributor could impact this interplay and affect the presentation of health disparities between populations. However, the relationship between these factors with genetic ancestry in Colombian women, a highly admixed population from Latin America, has not been explored. For that matter, we aimed to assess contributing factors to breast cancer health disparities according to genetic ancestry in Colombian patients from a national cancer reference center.

## Materials and methods

### Sample selection and data recollection

We collected formalin-fixed paraffin-embedded (FFPE) blocks from 361 patients with ductal invasive breast carcinomas diagnosed between 2013 and 2015 at the Colombian National Cancer Institute (NCI) in Bogotá D.C, a national reference center for cancer treatment that admits patients from all country regions.

Patients’ clinical-pathological data was collected from the hospital medical records system. This database also provides access to patients’ relevant sociodemographic information, including socioeconomic stratum and health insurance scheme. The socioeconomic stratum refers to Colombian social system where public services (water, gas, and electricity) are charged differentially to people according to the neighborhood they live in, reflecting individuals’ economic capacity. Typically, low-income families reside in I or II strata, middle-income families in III and IV, and high-income households in V or VI [19]. On the other hand, health insurance scheme refers to Colombia’s health system stratification, which dictates that all individuals must be covered under one of the two available health insurance schemes: a contributive scheme, financed by payroll contributions, and a subsidized scheme for people with minimal or no economic resources, financed by taxes and the government’s budget [20]. Within the contributive scheme, individuals can also be beneficiaries of a relative’s health. In that sense, both socioeconomic stratum and health insurance scheme serve as proxies for socioeconomic capacity. During data and FFPE collection, information on individual participants’ identification was available.

After collecting biological specimens, immunohistochemistry (IHC) was conducted and evaluated by a single pathologist to determine the expression of estrogen receptor (ER), progesterone receptor (PR), HER2, and Ki67 for breast cancer subtype classification, following the recommendations of the American Society of Clinical Oncology (ASCO)/College of American Pathologists (CAP) guidelines [21].

These samples were part of a previous study that aimed to analyze the association between genetic ancestry and ER/HER2/GRB7 status [22], and for this study, sample size was estimated based on the median fraction of IA ancestry for the Colombian population according to previous published data [23], using an absolute error of 5%. This research was approved by the Colombian NCI ethics committee and defined as risk-free, therefore, according to the Colombian laws, it was considered that no informed consent was required.

### Genetic ancestry estimation

DNA was extracted from non-tumoral FFPE blocks using the AllPrep DNA/RNA FFPE kit (Qiagen, Inc., Valencia, CA, USA) following the manufacturer’s protocol. Nucleic acid concentration was quantified by NanoDrop ND1000 Spectrophotometer (Thermo Scientific, Wilmington, USA). A panel of 106 Single Nucleotide Polymorphisms (SNPs) previously validated as Ancestry Informative Markers (AIMs) [24] were genotyped at the University of Minnesota Genomics Center, using the Sequenom technology. SNPs with a call rate <90% or that deviated from Hardy-Weinberg equilibrium were removed from the analysis, leaving 87 SNPs for individual genetic ancestry estimation. A total of 361 samples were genotyped and 308 remained after excluding samples with a genotype call rate <85%. We genotyped 10 duplicate pairs and the overall discordance rate was 0. Quality control of the genotyped data was performed in PLINK 1.9 [25], and the software Admixture 1.3 [26] was used under an admixture model (k = 3) to estimate IA, European and African ancestry fractions.

### Statistical analysis

We classified clinical-pathological and sociodemographic variables into the following groups: etiology-related factors (e.g., age of diagnosis), tumor-biology factors (e.g., tumor size), and socioeconomic factors (e.g., health insurance), each of which reflects potential sources of racial/ethnic breast cancer disparities. We also considered variables related to treatment administration (e.g., neoadjuvant therapy) as an indicator of disease management. Categorical variables were summarized as absolute and relative frequencies. We applied a Kruskal–Wallis test to assess differences in the European, IA and African ancestry fractions according to the distribution of each of the categorical variables. An additional analysis was conducted for genetic ancestry as a categorical variable (high vs. low according to the median), for which a Chi-squared (X^2^) test was applied. Lastly, a logistic regression model was used to evaluate the association between genetic ancestry and each of the defined contributing factors to health disparities. Unknown and not classifiable categories were not included in the statistical analyses. All statistical analyses were performed using the R-Studio software version 1.2.5019. Differences were considered statistically significant if *p*<0.05.

## Results

### Patients’ sociodemographic and clinical-pathological characteristics

Patients’ clinical-pathological and sociodemographic information is described in Table 1. Patients came mostly from Colombia’s capital city, Bogotá D.C (52.4%), and 8% came from other main cities around the country. A total of 143 patients (39.6%) came from little towns and more scattered places around the country, known as provinces, where there is less access to oncological services; consequently, these patients are referred to main cities, like Bogotá D.C, for health attention. The majority were either married or in free union (45.4%) and have had at least one child. As a socioeconomic status indicator, we used the socioeconomic stratum [19]. Most of breast cancer patients included in this study live in low-income neighborhoods (stratums I and II: 53.3%). This is consistent with the fact that only 13% of the study population had a technician or university education.

**Table 1:** Sociodemographic and clinical-pathological characteristics of the study population.

Characteristic	N (%)
**Sociodemographic characteristics**	
**Provenance region**	
Bogotá D.C (Colombia’s capital)	189 (52.4)
Other Colombian main cities	29 (8.0)
Town/provinces	143 (39.6)
**Socioeconomic stratum**	
Low (I/II)	193 (53.5)
Medium (III/IV)	56 (15.5)
High (V/VI)	3 (0.8)
Unknown	109 (30.2)
**Education level**	
Did not attend school	60 (16.6)
Elementary/Middle/High school	202 (56.0)
Technician school/University	47 (13.0)
Unknown	52 (14.4)
**Marital status**	
Married/Free union	164 (45.4)
Divorced	40 (11.1)
Single	98 (27.1)
Widowed	59 (16.3)
**Parity**	
0	29 (8.0)
1	61 (16.9)
>2	185 (51.2)
Unknown	86 (23.8)
**Health insurance**	
Contributive regime	175 (48.5)
Subsidized regime	176 (48.8)
Unknown	10 (2.8)
**Health insurance affiliation classification**	
Contributor	127 (35.2)
Beneficiary	47 (13.0)
Subsidized	176 (48.8)
Unknown	11 (3.0)
**Clinical-pathological characteristics**	
**Age of diagnosis**	
≤50	83 (23.0)
>50	278 (77.0)
**Breast cancer histological type**	
Ductal invasive carcinoma	100 (100.0)
**AJCC Clinical stage**	
I	40 (11.1)
II	156 (43.2)
III	158 (43.8)
IV	7 (1.9)
**Scarff-Bloom Richardson**	
I	30 (8.3)
II	192 (53.2)
III	138 (38.2)
Unknown	1 (0.3)
**Tumor size (mm)**	
≤ 20	96 (26.6)
21–49	146 (40.4)
≥ 50	107 (29.6)
Unknown	12 (3.3)
**Histological invasion**	
Negative	139 (38.5)
Positive	186 (51.5)
Unknown	36 (10.0)
**Lymph node involvement**	
Negative	108 (38.3)
Positive	173 (61.3)
Unknown	1 (0.4)
**Ki67 index**	
High (≥20%)	213 (59.0)
Low (<20%)	148 (41.0)
**Neoadjuvant treatment**	
Received	186 (51.5)
Did not receive	175 (48.5)
**Neoadjuvant treatment response**	
Complete	24 (6.6)
Partial	67 (18.6)
Stable	10 (2.8)
Progression	29 (8.0)
Unknown	56 (15.5)
**Breast cancer intrinsic subtype**	
Luminal/HER2-	236 (65.4)
Luminal/HER2+	37 (10.2)
HER2-enriched	21 (5.8)
Triple-negative	58 (16.1)
Not classifiable	9 (2.5)
**Recurrence**	
Negative	247 (68.4)
Positive	87 (24.1)
Unknown	27 (7.5)
**Vital state**	
Alive	277 (76.7)
Deceased	83 (23.0)
Unknown	1 (0.3)

Regarding Colombia’s available health insurance schemes, contributive and subsidized [20], we found that half of the patients included belonged to the latter group (48.8%). Moreover, among individuals within the contributive scheme, we observed that 13% of our patients are beneficiaries, leaving only 35.2% of the study population under a non-subsidized insurance model (Table 1).

Regarding clinical-pathological variables, most patients were diagnosed over the age of 50 (77.0%) with ductal invasive carcinomas (100%), at advanced clinical stages (AJCC Clinical stage III: 43.8% and IV: 1.9%), with moderately differentially tumors (Scarff-Bloom Richardson II: 53.2%) and sizes that ranged between 21–49 millimeters (mm) (40.4%). Furthermore, over half of the study population presented histological invasion (51.5%), lymph-node involvement (61.3%), and a high Ki67 index (≥20%: 59.0%) at diagnosis. Intrinsic subtype distribution showed that luminal/HER2- tumors were the most prevalent subtypes (65.4%), followed by the TN (16.1%), luminal/HER2+ (10.2%), and HER2-enriched subtypes (5.8%). At the end of the study, 24.1% of the patients had presented recurrence, and 23% had already died.

### Contributing factors to breast cancer health disparities according to genetic ancestry

**Fig 1:**
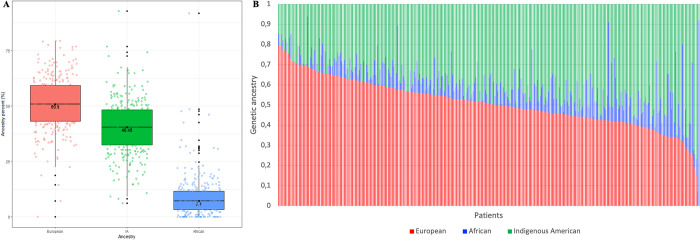
A. Median values of genetic ancestry (European, IA and African) among breast cancer patients from Colombia. B. Genetic ancestry distribution for 308 breast cancer patients from Colombia. Each patient is represented by a vertical bar at the x-axis.

Genetic ancestry data was available for 85.3% of the cases (308/361). The average genetic ancestry proportions for the European, IA, and African components were 50.9%, 40.6%, and 7.0%, respectively (Fig 1A). According to the 2018 Colombian census, almost 90% of the population self-identify as mestizo [27,28], which is consistent with our results where most of the patients included showed a highly admixed genetic profile, mainly between the European and the IA components (Fig 1B). We analyzed genetic ancestry as a continuous variable, in order to measure differences in genetic ancestry fractions according to different etiology, tumor-specific, treatment administration, and socioeconomic factors, all of which might contribute to breast cancer health disparities (Table 2).

**Table 2:** Distribution of European, IA, and African ancestry according to potential sources of breast cancer health disparities.

	Ancestry Category	N	European ancestry fraction	*p* value	IA ancestry fraction	*p* value	African ancestry fraction	*p* value
			(median [IQR])		(median [IQR])		(median [IQR])	
**Etiology factors**	**Age at diagnosis**							
	**≤50 years**	75	0.48 [0.42, 0.57]	0.224	0.42 [0.34, 0.49]	0.412	0.06 [0.03, 0.13]	0.670
	**>50 years**	233	0.51 [0.44, 0.60]		0.40 [0.32, 0.48]		0.07 [0.04, 0.11]	
	**Body mass index**							
	**≤25**	103	0.53 [0.45, 0.59]	0.150	0.38 [0.31, 0.46]	0.034	0.07 [0.03, 0.12]	0.693
	**>25**	204	0.49 [0.43, 0.60]		0.42 [0.34, 0.49]		0.07 [0.03, 0.11]	
**Tumor-specific factors**	**ER status**							
	**Negative**	74	0.52 [0.43, 0.58]	0.939	0.42 [0.33, 0.48]	0.778	0.06 [0.04, 0.11]	0.876
	**Positive**	234	0.50 [0.43, 0.59]		0.40 [0.32, 0.48]		0.07 [0.03, 0.12]	
	**PR status**							
	**Negative**	101	0.52 [0.44, 0.58]	0.823	0.41 [0.34, 0.47]	0.813	0.07 [0.04, 0.11]	0.986
	**Positive**	207	0.50 [0.43, 0.60]		0.40 [0.31, 0.49]		0.07 [0.03, 0.12]	
	HER2 status*							
	**Negative (0+/1+)**	236	0.50 [0.43, 0.58]	0.734	0.41 [0.33, 0.48]	0.428	0.07 [0.03, 0.12]	0.761
	**Positive (3+)**	54	0.51 [0.42, 0.58]		0.42 [0.36, 0.49]		0.06 [0.03, 0.12]	
	Intrinsic subtype*							
	**Luminal/HER2-**	199	0.50 [0.44, 0.59]	0.363	0.39 [0.32, 0.48]	0.457	0.07 [0.03, 0.11]	0.923
	**Luminal/HER2+**	30	0.46 [0.42, 0.56]		0.43 [0.38, 0.50]		0.07 [0.03, 0.15]	
	**HER2-enriched**	19	0.53 [0.43, 0.64]		0.42 [0.30, 0.48]		0.06 [0.04, 0.10]	
	**Triple negative**	52	0.51 [0.42, 0.56]		0.42 [0.33, 0.49]		0.07 [0.04, 0.12]	
	**AJCC Clinical stage**							
	**I/II**	173	0.51 [0.44, 0.61]	0.125	0.40 [0.32, 0.48]	0.942	0.06 [0.02, 0.10]	0.036
	**III/IV**	135	0.50 [0.42, 0.58]		0.41 [0.33, 0.48]		0.08 [0.04, 0.12]	
	**Tumor size**							
	**≤20 mm**	84	0.54 [0.47, 0.62]	0.009	0.39 [0.29, 0.45]	0.022	0.07 [0.05, 0.11]	0.345
	**>20 mm**	215	0.50 [0.42, 0.58]		0.42 [0.33, 0.50]		0.06 [0.03, 0.12]	
**Treatment-related factors**	**Neoadjuvant treatment**							
	**Received**	155	0.48 [0.42, 0.56]	0.002	0.42 [0.34, 0.50]	0.055	0.07 [0.03, 0.12]	0.191
	**Did not receive**	153	0.53 [0.45, 0.62]		0.40 [0.30, 0.47]		0.07 [0.03, 0.11]	
	**Neoadjuvant treatment response**							
	**Complete**	20	0.43 [0.41, 0.52]	0.133	0.43 [0.34, 0.50]	0.576	0.12 [0.06, 0.15]	0.006
	**Partial**	54	0.47 [0.42, 0.56]		0.43 [0.35, 0.51]		0.05 [0.02, 0.10]	
	**Stable**	9	0.45 [0.39, 0.52]		0.43 [0.38, 0.45]		0.10 [0.09, 0.12]	
	**Progression**	26	0.54 [0.44, 0.62]		0.41 [0.30, 0.43]		0.06 [0.03, 0.10]	
	**Surgical management**							
	**Quadrantectomy**	141	0.52 [0.45, 0.61]	0.075	0.40 [0.31, 0.47]	0.155	0.07 [0.03, 0.12]	0.900
	**Mastectomy**	167	0.50 [0.42, 0.58]		0.42 [0.33, 0.50]		0.07 [0.03, 0.11]	
**Socioeconomic factors**	**Education level**							
	**Did not attend school**	47	0.50 [0.44, 0.61]	0.567	0.38 [0.34, 0.47]	0.169	0.07 [0.04, 0.11]	0.060
	**Middle/high school**	174	0.50 [0.43, 0.58]		0.42 [0.33, 0.49]		0.07 [0.03, 0.11]	
	**Tech/University**	43	0.53 [0.46, 0.61]		0.37 [0.30, 0.43]		0.10 [0.06, 0.14]	
	**Socioeconomic stratum**							
	**Low (I-II)**	162	0.49 [0.42, 0.58]	0.047	0.42 [0.34, 0.49]	0.097	0.07 [0.03, 0.12]	0.924
	**Medium (III-IV)**	50	0.55 [0.45, 0.62]		0.37 [0.30, 0.47]		0.07 [0.05, 0.11]	
	**Insurance regime**							
	**Contributory**	147	0.53 [0.46, 0.61]	<0.001	0.39 [0.31, 0.47]	0.017	0.07 [0.03, 0.12]	0.781
	**Subsidized**	152	0.48 [0.42, 0.55]		0.42 [0.34, 0.50]		0.07 [0.03, 0.11]	
	**Provenance region**							
	**Urban area**	182	0.50 [0.43, 0.57]	0.292	0.40 [0.34, 0.48]	0.675	0.07 [0.03, 0.12]	0.766
	**Rural area**	126	0.51 [0.44, 0.62]		0.41 [0.30, 0.49]		0.07 [0.03, 0.11]	
	**Civil status**							
	**Married/civil union**	140	0.49 [0.42, 0.58]	0.067	0.42 [0.34, 0.50]	0.099	0.07 [0.03, 0.12]	0.524
	**Single (widow, divorced, single)**	168	0.52 [0.44, 0.60]		0.39 [0.31, 0.47]		0.07 [0.03, 0.10]	
	**Parity (No. Children)**							
	**0**	24	0.53 [0.48, 0.60]	0.048	0.37 [0.32, 0.44]	0.078	0.05 [0.03, 0.11]	0.674
	**1**	51	0.47 [0.42, 0.55]		0.45 [0.36, 0.52]		0.07 [0.05, 0.11]	
	**≥2**	158	0.52 [0.44, 0.60]		0.40 [0.31, 0.48]		0.07 [0.03, 0.12]	

IA: Indigenous American; IQR: Interquartile range; ER: Estrogen receptor; PR: Progesterone receptor.

*HER2 equivocal (2+) cases with no confirmatory result (n = 22) and not classifiable subtypes (n = 9) were excluded from the analyses.

Among etiology-related factors, such as age of diagnosis and body mass index (BMI), we observed a higher median of IA ancestry fraction in overweight patients compared to the lean group (>25: 0.42 vs. ≤25: 0.38, respectively, *p* = 0.034). No statistically significant differences in genetic ancestry fractions were observed by age at diagnosis. Regarding tumor-specific factors, a higher median of African ancestry was observed in patients diagnosed at higher clinical stages (III/IV: 0.08 vs. I/II: 0.06, *p* = 0.036). On the other hand, patients with larger tumors (>20 mm) presented a lower median of European ancestry (0.50 vs. 0.54, *p* = 0.009) and a higher IA| ancestry fraction (0.42 vs. 0.39, *p* = 0.022), compared to patients with smaller tumors (≤ 20 mm). No statistically significant differences in any of the genetic ancestry fractions were found according to ER, PR, HER2 status, and intrinsic subtype.

Concerning treatment-related factors, a lower median of European ancestry fraction and a tendency for higher IA ancestry was observed in patients who received neoadjuvant therapy, compared to patients who did not receive this treatment (for European ancestry: 0.48 vs. 0.53, respectively, *p* = 0.002; for IA ancestry: 0.42 vs. 0.40, respectively, *p* = 0.055). Interestingly, a significantly higher African ancestry was observed in patients who achieved a complete clinical or pathological response (0.12) or presented a stable clinical response (0.10), compared to patients who either progressed (0.06) or had a partial response (0.05) to neoadjuvant treatment (*p* = 0.006). In addition, a higher European ancestry fraction was observed in patients who underwent conservative surgeries, compared to patients who received mastectomies; however, this difference did not reach statistically significant values (0.52 vs. 0.50, *p* = 0.075).

Regarding socioeconomic and demographic factors, our results showed that patients who live in middle-income households (III/IV stratums) present higher European ancestry levels compared to patients in the low stratum group (0.55 vs. 0.49, *p* = 0.047); patients in high-income stratums were not included in the analysis given the low number of samples in this group (n = 3). Likewise, significantly higher European ancestry (0.53 vs. 0.48, *p*<0.001) and lower IA median ancestry (0.39 vs. 0.42, *p* = 0.017) were observed in patients from the contributory insurance regime, compared to patients from the subsidized scheme. Lastly, we also observed higher European ancestry fractions in patients that had never had children before (0.53), compared to patients with one (0.47) or more than 2 children (0.52) (*p* = 0.048). No statistically significant differences in genetic ancestry fractions were found according to civil status, educational level, and provenance region. We conducted a second analysis where genetic ancestry was categorized as high and low, according to the median value, and found similar results (S1 Table).

Furthermore, a regression analysis showed an association between a higher IA ancestry and being overweight (BMI>25: OR = 1.73, 95% CI, 1.05–2.91, *p* = 0.0321), having larger tumors (>20 mm: OR = 1.91, 95% CI, 1.13–3.32, *p* = 0.0173), and being in a subsidized insurance regime (OR = 1.73, 95% CI, 1.07–2.85, *p* = 0.0254). On the other hand, patients with higher European ancestry showed lower odds for larger tumors (≥20 mm: OR = 0.45, 95% CI, 0.25–0.77, *p* = 0.00496), for receiving neoadjuvant therapy (OR = 0.49, 95% CI, 0.30–0.80, *p* = 0.00487), and being in a subsidized health regime (OR = 0.42, 95% CI, 0.25–0.69, *p* = 0.0008). At the same time, higher odds were observed in patients with higher European ancestry fractions to live in higher socioeconomic stratums (III/IV: OR = 1.93, 95% CI, 0.99–3.87, *p* = 0.055).

## Discussion

It has been observed that individuals among population groups not only share a heritage and many cultural traditions, but they also seem to have comparable socioeconomic conditions, such as similar monetary income and education level [29,30]. It is well known that socioeconomic features are well-established contributing factors to health disparities, nonetheless, biological factors can also contribute to the differences reported in incidence, prevalence, and mortality among these population groups [12,31,32]. High genomic resolution studies have identified disease-related loci enriched for a particular ancestry component in the genome of highly admixed populations from Mexico [33], Puerto Rico [34], and Colombia [35]. These investigations have led to the hypothesis that the genetic footprint left by the colonization process in Latin America might contribute to health disparities among admixed populations, affecting several health determinants and epidemiological measures such as incidence, prevalence, and mortality [35].

In this study, we focused on genetic ancestry as one of the potential biological contributors to health disparities, and assessed its relationship with important socioeconomic factors, but also with other etiologic, tumor, and treatment-administration-related factors, in a cohort of breast cancer patients from Colombia. In terms of genetic complexity, Latin American countries like Colombia are of great interest, as a result of the genetic admixture that began in the Americas with the arrival of the Spanish colonization and trans-Atlantic slave trade around 500 years ago [36]. This process entailed a massive genetic exchange between three continental groups (Europeans, Native Americans, and Africans) that until that point, had never been in contact before. This brought a particularly high level of genetic diversity among Latin American countries, including Colombia [35].

Unlike the United States (US) population, Colombia counts with just a few officially recognized minority ethnic groups, including the Indígenas, Roms, Raizales, Palanqueros, and Afro-Colombians. However, according to the 2018 Colombian census, almost 90% of the people do not self-identify with any of these ethnic groups, and instead, self-identify as mestizos [27,28]. As reported by Chande et al [37], these ethnic categories are strongly correlated with genetic ancestry, but they do not capture the complexity of ancestry and admixture seen in the Colombian population. Therefore, studying health disparities-contributing factors according to ethnic groups is not useful in a country like Colombia. Instead, we evaluated genetic ancestry and found consistent associations with what has been reported before in other population groups [38,39].

Our results showed that patients with higher European ancestry presented smaller tumors, received less neoadjuvant therapy, and belonged to higher socioeconomic stratums. In contrast, patients with higher IA ancestry fractions were more frequently classified as obese, presented larger tumors, and received neoadjuvant chemotherapy. A previous study by Fejerman et al. [40], which investigated the association between genetic ancestry and clinical outcome in U.S. Latina women with breast cancer, hypothesized that the relationship between genetic ancestry and this clinical feature might be related to the strong association between self-identified ethnicity and socioeconomic status [40]. Epidemiology studies conducted in the US have reported that self-identified White people, who are primarily of European descent, often have a higher socioeconomic status, whereas other population groups like African Americans, with a greater African ancestry component, and Latinos, with an important IA ancestry fraction in their genome, allegedly live under inferior socioeconomic conditions [41,42]. For example, a study conducted in Mexican and Colombian patients with type-2 diabetes reported a positive association between IA ancestry and lower socioeconomic stratums [43]. Certainly, having a low socioeconomic status will lead to fewer educational opportunities, along with less healthy lifestyle habits and lower access and awareness to screening programs [44,45], which will finally correlate to the presentation of bad prognosis breast cancer clinical-pathological features [46,47].

Furthermore, a higher proportion of women with higher IA ancestry was found among the subsidized health insurance group, whereas a greater European ancestry fraction was observed for the contributory health insurance scheme. Reports for gastric cancer in Colombia have stated that patients in the subsidized regime have lower survival times and a higher mortality risk, compared to patients in the contributory regime, as well as those affiliated to the special insurance scheme [48]; this corresponds to a special program for government workers in the fields of education, military, policy, and petrochemical industry [20]. Likewise, an extensive review of the latest annuals reports from the National Health Institute of Colombia stated that patients in the contributory regime often present cancer at early stages at diagnosis, compared to those affiliated to the subsidized regime [49]. It is likely that this might be related to the better healthcare services provided by the contributory and special regimes, however, other reports have found that, regardless of their type of insurance affiliation, patients from higher socioeconomic stratums present better survival probabilities [48]. Understanding the role of these health determinants in the context of breast cancer, a multifactorial disease, can be challenging, as these variables are often highly correlated with each other. In that sense, we hypothesized that having better healthcare services and other aspects related to living in a higher socioeconomic stratum, like a healthy diet and a higher education level, might explain why in our study, patients with higher European ancestry presented better clinical-pathological features, and also, that women with higher IA ancestry presented less favorable clinical-pathological traits like obesity and larger tumors.

On the other hand, higher African ancestry fractions were found among patients with poor clinical-pathological features, like advanced clinical stages and larger tumors. Several studies in the US have reported these associations, claiming that this could be due to both socioeconomic and genetic factors [50–52]. The latter includes the presence of population-specific genetic variants that predispose women with high African ancestry to develop more aggressive breast cancer characteristics and intrinsic subtypes like the TN [12,52,53]. However, in our study, we did not find a statistically significant association between a higher fraction of African ancestry and a greater prevalence of TN tumors. This might be possibly related to the lower contribution of this particular component within Colombian patients. Compared to studies where this association has been reported [54,55], the African ancestry contribution in our study is considerably low, at approximately 7%. This poses a challenge in drawing conclusions regarding the potential contributions of the African component to disease phenotype and to breast cancer health disparities in Colombian women.

We certainly encountered some limitations during the conduction of this study. The majority of the patients’ socioeconomic data was retrieved from medical records and government sources, however, there were still many cases with missing information, especially for socioeconomic stratum and education level, which were some of the most relevant variables explored in the analyses. Along the same line, almost all the patients that are admitted at the Colombian NCI are from the low and medium socioeconomic stratums; just 3 of our patients belonged to the high (V and VI) category. It is possible that this fact did not allow us to detect even further differences between socioeconomic stratums by genetic ancestry. Other limitations include the small sample size among several categories and the generally low contribution of the African ancestry component among the included patients. It is possible that this might have reduced the study’s statistical power and limited the opportunity to find biological associations. We also highlight the potential impact on AIMs genotyping quality resulting from the use of FFPE blocks for DNA extraction and genetic ancestry estimation, as these samples are known to be difficult to handle due to their high degree of nucleic acid degradation [56]. Even so, our study also had several strengths. All cases were recruited from a single institution, whereby, there were no inclusion biases. In addition, a single pathologist assessed all immunohistochemistry biomarkers, and a single person collected clinical and pathological information from medical records, all of which contributed to the homogeneity of our data.

Our results show important differences in several aspects of the disease according to genetic markers. Considering the current Colombian Health Ministry guidelines for breast cancer screening and early detection [57], our work could potentially be used to implement a more guided and personalized approach for future breast cancer screening and management protocols in the country. For instance, health determinants and genetic biomarkers, such as genetic ancestry, could potentially be incorporated as covariates in future artificial intelligence models to enhance the decision-making process in clinical settings.

## Conclusions

Overall, our findings show that patients with higher IA ancestry present clinical-pathological characteristics of poor prognosis like obesity and larger tumors and are more frequently affiliated to a subsidized healthcare regime. Furthermore, we found that patients with higher European ancestry present smaller tumors, do not receive as much neoadjuvant chemotherapy, and usually belong to higher socioeconomic stratums and to the contributory healthcare regime. Our results reflect complex interactions between socioeconomic, etiological, tumor-related, and genetic factors, that ultimately affect the presentation of health disparities reported in breast cancer.

## Supporting information

S1 Table Distribution of potential sources of breast cancer health disparities according to genetic ancestry status (high vs. low according to the median). IA: Indigenous American; ER: Estrogen receptor; PR: Progesterone receptor. *HER2 equivocal (2+) cases with no confirmatory result (n = 22) and not classifiable subtypes (n = 9) were excluded from the analyses.
(DOCX)
