# Supplementary material for: Genetic ancestry is related to potential sources of breast cancer health disparities among Colombian women
Source: PLoS One. 2024 Jun 27;19(6):e0306037. doi: 10.1371/journal.pone.0306037 (PMC11210782; doi:10.1371/journal.pone.0306037)
Supplement: S1 Table — IA: Indigenous American; ER: Estrogen receptor; PR: Progesterone receptor. *HER2 equivocal (2+) cases with no confirmatory result (n = 22) and not classifiable subtypes (n = 9) were excluded from the analyses. (DOCX) [file pone.0306037.s001.docx]

**S1 Table. Distribution of potential sources of breast cancer health disparities according to genetic ancestry status (high vs. low according to the median).**

|  | **Ancestry**  **Category** | **N** | **European ancestry fraction** | | ***p* value** | **IA ancestry fraction** | | ***p* value** | **African ancestry fraction** | | ***p* value** |
| --- | --- | --- | --- | --- | --- | --- | --- | --- | --- | --- | --- |
|  |  |  | **Low (≤0.5091)**  **N (%)** | **High (>0.5091)**  **N (%)** |  | **Low (≤0.4046)**  **N (%)** | **High (>0.4046)**  **N (%)** |  | **Low (≤0.0708)**  **N (%)** | **High (>0.0708)**  **N (%)** |  |
| **Etiology factors** | **Age at diagnosis** | | | | | | | | | | |
|  | **≤50 years** | 75 | 42 (27.3) | 33 (21.4) | 0.288 | 35 (22.7) | 40 (26.0) | 0.595 | 42 (27.3) | 33 (21.4) | 0.288 |
|  | **>50 years** | 233 | 112 (72.7) | 121 (78.6) |  | 119 (77.3) | 114 (74.0) |  | 112 (72.7) | 121 (78.6) |  |
|  | **Body mass index** | | | | | | | | | | |
|  | **≤25** | 103 | 40 (26.0) | 63 (41.2) | 0.007 | 59 (38.6) | 44 (28.6) | 0.083 | 51 (33.1) | 52 (34.0) | 0.968 |
|  | **>25** | 204 | 114 (74.0) | 90 (58.8) |  | 94 (61.4) | 110 (71.4) |  | 103 (66.9) | 101 (66.0) |  |
| **Tumor-specific factors** | **ER status** | | | | | | | | | | |
|  | **Negative** | 74 | 33 (21.4) | 41 (26.6) | 0.351 | 35 (22.7) | 39 (25.3) | 0.689 | 40 (26.0) | 34 (22.1) | 0.505 |
|  | **Positive** | 234 | 121 (78.6) | 113 (73.4) |  | 119 (77.3) | 115 (74.7) |  | 114 (74.0) | 120 (77.9) |  |
|  | **PR status** | | | | | | | | | | |
|  | **Negative** | 101 | 47 (30.5) | 54 (35.1) | 0.466 | 48 (31.2) | 53 (34.4) | 0.627 | 53 (34.4) | 48 (31.2) | 0.627 |
|  | **Positive** | 207 | 107 (69.5) | 100 (64.9) |  | 106 (68.8) | 101 (65.6) |  | 101 (65.6) | 106 (68.8) |  |
|  | **HER2 status*** | | | | | | | | | | |
|  | **Negative (0+/1+)** | 236 | 124 (82.1) | 112 (80.6) | 0.852 | 118 (83.1) | 118 (79.7) | 0.558 | 115 (79.3) | 121 (83.4) | 0.451 |
|  | **Positive (3+)** | 54 | 27 (17.9) | 27 (19.4) |  | 24 (16.9) | 30 (20.3) |  | 30 (20.7) | 24 (16.6) |  |
|  | **Intrinsic subtype*** | | | | | | | | | | |
|  | **Luminal/HER2-** | 199 | 102 (66.7) | 97 (66.0) | 0.316 | 105 (70.9) | 94 (61.8) | 0.329 | 94 (62.7) | 105 (70.0) | 0.469 |
|  | **Luminal/HER2+** | 30 | 19 (12.4) | 11 ( 7.5) |  | 11 ( 7.4) | 19 (12.5) |  | 17 (11.3) | 13 ( 8.7) |  |
|  | **HER2-enriched** | 19 | 7 ( 4.6) | 12 ( 8.2) |  | 9 ( 6.1) | 10 ( 6.6) |  | 12 ( 8.0) | 7 ( 4.7) |  |
|  | **Triple negative** | 52 | 25 (16.3) | 27 (18.4) |  | 23 (15.5) | 29 (19.1) |  | 27 (18.0) | 25 (16.7) |  |
|  | **AJCC Clinical stage** | | | | | | | | | | |
|  | **I/II** | 173 | 82 (53.2) | 91 (59.1) | 0.358 | 89 (57.8) | 84 (54.5) | 0.646 | 94 (61.0) | 79 (51.3) | 0.108 |
|  | **III/IV** | 135 | 72 (46.8) | 63 (40.9) |  | 65 (42.2) | 70 (45.5) |  | 60 (39.0) | 75 (48.7) |  |
|  | **Tumor size** | | | | | | | | | | |
|  | **≤20 mm** | 84 | 34 (23.0) | 50 (33.1) | 0.069 | 49 (32.9) | 35 (23.3) | 0.087 | 36 (23.5) | 48 (32.9) | 0.095 |
|  | **>20 mm** | 215 | 114 (77.0) | 101 (66.9) |  | 100 (67.1) | 115 (76.7) |  | 117 (76.5) | 98 (67.1) |  |
| **Treatment-related factors** | **Neoadjuvant treatment** | | | | | | | | | | |
|  | **Received** | 155 | 89 (57.8) | 66 (42.9) | 0.012 | 70 (45.5) | 85 (55.2) | 0.111 | 72 (46.8) | 83 (53.9) | 0.254 |
|  | **Did not receive** | 153 | 65 (42.2) | 88 (57.1) |  | 84 (54.5) | 69 (44.8) |  | 82 (53.2) | 71 (46.1) |  |
|  | **Neoadjuvant treatment response** | | | | | | | | | | |
|  | **Complete** | 20 | 14 (22.6) | 6 (12.8) | 0.140 | 7 (14.6) | 13 (21.3) | 0.844 | 6 (11.5) | 14 (24.6) | 0.021 |
|  | **Partial** | 54 | 32 (51.6) | 22 (46.8) |  | 25 (52.1) | 29 (47.5) |  | 31 (59.6) | 23 (40.4) |  |
|  | **Stable** | 9 | 6 ( 9.7) | 3 ( 6.4) |  | 4 ( 8.3) | 5 ( 8.2) |  | 1 ( 1.9) | 8 (14.0) |  |
|  | **Progression** | 26 | 10 (16.1) | 16 (34.0) |  | 12 (25.0) | 14 (23.0) |  | 14 (26.9) | 12 (21.1) |  |
|  | **Surgical management** | | | | | | | | | | |
|  | **Quadrantectomy** | 141 | 66 (42.9) | 75 (48.7) | 0.360 | 78 (50.6) | 63 (40.9) | 0.109 | 72 (46.8) | 69 (44.8) | 0.819 |
|  | **Mastectomy** | 167 | 88 (57.1) | 79 (51.3) |  | 76 (49.4) | 91 (59.1) |  | 82 (53.2) | 85 (55.2) |  |
| **Socioeconomic factors** | **Education level** | | | | | | | | | | |
|  | **Did not attend school** | 47 | 25 (18.8) | 22 (16.8) | 0.467 | 26 (19.3) | 21 (16.3) | 0.034 | 25 (19.4) | 22 (16.3) | 0.028 |
|  | **Middle/high school** | 174 | 90 (67.7) | 84 (64.1) |  | 80 (59.3) | 94 (72.9) |  | 91 (70.5) | 83 (61.5) |  |
|  | **Tech/University** | 43 | 18 (13.5) | 25 (19.1) |  | 29 (21.5) | 14 (10.9) |  | 13 (10.1) | 30 (22.2) |  |
|  | **Socioeconomic stratum** | | | | | | | | | | |
|  | **Low (I-II)** | 162 | 90 (81.1) | 72 (71.3) | 0.130 | 75 (71.4) | 87 (81.3) | 0.125 | 80 (75.5) | 82 (77.4) | 0.871 |
|  | **Medium (III-IV)** | 50 | 21 (18.9) | 29 (28.7) |  | 30 (28.6) | 20 (18.7) |  | 26 (24.5) | 24 (22.6) |  |
|  | **Insurance regime** | | | | | | | | | | |
|  | **Contributory** | 147 | 62 (41.1) | 85 (57.4) | 0.007 | 84 (56.8) | 63 (41.7) | 0.013 | 72 (48.3) | 75 (50.0) | 0.861 |
|  | **Subsidized** | 152 | 89 (58.9) | 63 (42.6) |  | 64 (43.2) | 88 (58.3) |  | 77 (51.7) | 75 (50.0) |  |
|  | **Provenance region** | | | | | | | | | | |
|  | **Urban area** | 182 | 67 (43.5) | 59 (38.3) | 0.417 | 90 (58.4) | 92 (59.7) | 0.908 | 64 (41.6) | 62 (40.3) | 0.908 |
|  | **Rural area** | 126 | 87 (56.5) | 95 (61.7) |  | 64 (41.6) | 62 (40.3) |  | 90 (58.4) | 92 (59.7) |  |
|  | **Civil status** | | | | | | | | | | |
|  | **Married/civil union** | 140 | 76 (49.4) | 64 (41.6) | 0.208 | 63 (40.9) | 77 (50.0) | 0.137 | 66 (42.9) | 74 (48.1) | 0.423 |
|  | **Single (widow, divorced, single)** | 168 | 78 (50.6) | 90 (58.4) |  | 91 (59.1) | 77 (50.0) |  | 88 (57.1) | 80 (51.9) |  |
|  | **Parity (No. Children)** | | | | | | | | | | |
|  | **0** | 24 | 8 ( 6.8) | 16 (13.8) | 0.026 | 14 (12.2) | 10 ( 8.5) | 0.452 | 13 (11.4) | 11 ( 9.2) | 0.282 |
|  | **1** | 51 | 33 (28.2) | 18 (15.5) |  | 22 (19.1) | 29 (24.6) |  | 20 (17.5) | 31 (26.1) |  |
|  | **≥2** | 158 | 76 (65.0) | 82 (70.7) |  | 79 (68.7) | 79 (66.9) |  | 81 (71.1) | 77 (64.7) |  |

IA: indigenous American; ER: estrogen receptor; PR: progesterone receptor.

*HER2 equivocal (2+) cases with no confirmatory result (n=22) and not classifiable subtypes (n=9) were excluded from the analysis.
